# Data-independent proteome analysis of ARPE-19 cells

**DOI:** 10.1016/j.dib.2018.06.103

**Published:** 2018-07-04

**Authors:** Diwa Koirala, Sarka Beranova-Giorgianni, Francesco Giorgianni

**Affiliations:** University of Tennessee Health Science Center, Department of Pharmaceutical Sciences, 881 Madison Av., Pharmacy Bldg., Memphis, TN 38163, United States

## Abstract

We have performed a proteomics analysis of a human retinal pigment epithelial cell line (ARPE-19), which represents a widely used model for *in vitro* studies of cellular and molecular mechanisms related to human RPE cells (Dunn et al., 1996; Weigel et al., 2002) [1], [2]. Whole cell protein extracts were separated in four gel fractions via short (10 min) SDS-PAGE runs. Following fractionation and trypsin digestion, the resulting peptides were separated on a nano UPLC LC system and analyzed on-line with a QTof-IMS instrument: a tandem mass spectrometer with ion mobility separation (Synapt G2-Si). Data were acquired in data-independent mode (UDMS^E^), which allows for absolute and/or relative post-acquisition protein quantification (Silva et al., 2006) [3]. The proteome profile data obtained from this study can be used as a protein reference database with qualitative and quantitative protein information related to ARPE-19 cells under normal growth conditions.

**Specifications Table**

**Table:** 

Subject area	*Biology*
More specific subject area	*System biology of a human cell model; proteomics*
Type of data	*LC–MS/MS data*
How data was acquired	*Data-independent acquisition (UDMS*^*E*^*) using Waters Synapt G2-Si*
Data format	*Raw*
Experimental factors	*ARPE-19 cells were cultured to confluence under standard growth conditions. Cell whole protein content was extracted, fractionated by short SDS-PAGE and digested with trypsin. Peptides samples were analyzed by LC–MS/MS and the data analyzed by a label-free proteomics method*
Experimental features	*ARPE-19 cell whole proteome fractionated and characterized by bottom-up proteomics*
Data source location	*Dept. of Pharmaceutical Sciences, UTHSC - Memphis, TN, USA*
Data accessibility	http://www.ebi.ac.uk/pride/archive/projects/PXD009607
Related research article	*N/A*

**Value of the data**

• The data represent a large qualitative and quantitative map of proteins expressed in ARPE-19 cells.
• The data could be used as a proteome baseline to detect changes in the proteome profile as a result of environmental challenges, e.g. exposure to oxidative stress –inducing agents.
• The data could be used to develop ARPE-19 specific peptide spectral libraries for targeted proteomics studies.

## 1 Data

This data represents a label-free, data-independent mapping of the proteome of a human cell line: ARPE-19. This cell line is widely used as a model system to study the biology and function of the retinal pigment epithelium (RPE) [1], [2]. The cells were grown to confluence under normal growth conditions before harvesting and processing. The dataset relates to a total of 24 sample runs: six ARPE-19 biological replicates, 4 gel fractions per biological replicate.

## 2 Experimental design, materials, and methods

### 2.1 Cell cultures

The immortalized human retinal pigment epithelium cells ARPE-19 were purchased from the American Type Culture Collection (ATCC, Manassas, USA) and maintained in DMEM-F12 medium (ATCC, Manassas, USA) containing 2 mM L-glutamine supplemented with 10% fetal bovine serum (ATCC, Manassas, USA) and 100 μg/ml (Invitrogen, Carlsbad, USA) in a humidified atmosphere with 5% CO_2_ at 37 °C. After reaching confluence, the cells were washed once with phosphate buffered saline (PBS) and removed from the cell culture plate with a cell scraper. The collected cells were centrifuged for 5 min at 150×*g* in a refrigerated centrifuge and the resulting cell pellets stored in liquid nitrogen until further processing.

### 2.2 Short SDS-PAGE

Immediately before analysis, the cells were thawed and collected by a brief centrifugation. Cell pellets were directly re-suspended in 50 µl of RIPA buffer. Protein concentration was measured with Pierce micro BCA kit (Thermofisher) with BSA as an external protein standard. Thirty microliters of cell extract (*ca* 60 µg total protein) were mixed with 10 µl 4X sample loading buffer and 2 µl 20X reducing solution and boiled at 95 °C for 10 min. The denatured and reduced protein solutions were centrifuged at 20,000×*g* for 10 min and the supernatants loaded onto a 4–12% Bis-Tris gradient precast gel (Criterion XT, Biorad). The samples were run at 100 V constant voltage for 10 min. Following separation, the gels were stained with Coomassie Blue. Each sample lane was cut into 4 gel slices of equal size.

### 2.3 Trypsin digestion and peptide extraction

Gel slices were de-stained with a solution of 50 mM ammonium bicarbonate (ABC) in 50% acetonitrile-water by vortexing for 10 min. This step was repeated 3 times to remove all dye. For protein disulfide bond reduction and cysteine alkylation, a solution of 50 mM dithiothreitol (DTT) in 200 mM ABC was added to each gel slice, and the samples were incubated for 1 h at 56 °C. After removal of the reducing solution, the proteins were alkylated with a solution of 110 mM iodoacetamide in 200 ABC and incubation for 45 min at r.t. in the dark. Gel slices were washed with 200 mM ABC, dehydrated with 100 % acetonitrile (3 times) and dried in a vacuum centrifuge for 30 min. The gel slices were re-hydrated with trypsin digestion buffer containing 1.0 µg of sequencing grade trypsin (Promega) in 50 mM ABC and incubated O/N at 37 °C. The digest supernatants were collected and the residual peptides were extracted from the gel slices with a solution of 60% acetonitrile-35% water-5% TFA. The combined supernatants and extracted solutions were dried in a vacuum centrifuge. The dried peptide samples were reconstituted with 20 µl of a solution of water-0.1% TFA and desalted with ZipTip C18 micro-columns (Millipore) according to manufacturer’s procedure. Peptides were eluted from the ZipTip with 8 µl of 50% acetonitrile-50% water-0.1% TFA and mixed with 12 µl of water-0.5% acetic acid.

### 2.4 Data-independent acquisition

Desalted peptide solutions were mixed in a 1:1 (v:v) ratio with a 25 fmol/µl solution of yeast aldehyde dehydrogenase 1 (ADH) tryptic digest standard (Waters Corporation). Samples were analyzed on a Acquity UPLC M-Class nano-LC system (Waters Corporation) interfaced to a Quadrupole Time-of-flight (QTof) tandem mass spectrometer with ion mobility separation (IMS) (Synapt G2-Si, Waters Corporation). The LC–MS/MS system was operated via the MassLynx control software suite (v. 4.1). Samples were applied to an on-line LC pre-column trap by partial loop injection (4 µl) with the aid of the autosampler component of the Acquity M-class system. Peptides were eluted from the pre-column trap (Symmetry C18, 100 Å 5 µm, 180 µm × 20 mm, Waters Corporation) and separated on a nano flow UPLC column (HSS T3 1.8 µm, 75 µm × 250 mm, Waters Corporation) with a 90 min linear gradient of 2–40% mobile phase B (acetonitrile 0.1 % formic acid) and mobile phase A (water, 0.1 % formic acid). The eluted peptides were introduced into the mass spectrometer on-line via a nano electrospray source with temperature set at 80 °C and capillary voltage of 2.8 kV. The mass spectrometer quadrupole was set to efficiently transmit ions with *m*/*z* > 300 and the Tof to detect ions in the 50–2000 *m*/*z* range. The Glu-fibrinogen peptide was infused from the fluidics system of the instrument for lock-mass correction. The acquisition method included ion mobility separation, which preceded peptide parent ion fragmentation in the transfer cell. The instrument was set to allow the collision energy to cycle between low and high values in order to generate full-scan MS and fragment MS/MS spectra without parent ion isolation, which represents a data-independent MS/MS acquisition mode referred to as MS^E^ [4], or HDMS^E^ (high-definition MS^E^) if combined with ion mobility separation. The low collision energy was set at 6 V and the high collision energy was set to be drift time-specific and ramped during acquisition from 17 V to 60 V . This setting was implemented with the aid of a look up table included in the acquisition method and developed by modification of the procedure originally published by Distler et al and termed ultra-definition MS^E^ (UDMS^E^) [5].

### 2.5 Data analysis

The UDMS^E^ raw data were analyzed with Progenesis QI for proteomics (Nonlinear Dynamics) software platform. Peptides were identified with the “ion accounting” identification method from searching a non-redundant human database, which contained *ca* 20,000 protein entries. The search allowed for a maximum of one trypsin missed peptide cleavage, static modification of cysteine (carbamidomethylation), and variable modifications of methionine (oxidation) and asparagine and glutamine (deamidation). The peptide false discovery rate was set to less than 4%. Identified proteins were displayed according to the protein grouping method, which is a based on a less stringent application of the parsimony principle. The protein grouping method hides from the final protein count all proteins that share peptide matches with a “lead” protein, i.e., all proteins with lower coverage than the protein with the highest sequence coverage. However, the hidden proteins are not permanently deleted in the final output file and are available for further analysis. The identified proteins were quantified in Progenesis QI for proteomics with the “Hi-3” method [3] using yeast aldehyde dehydrogenase 1 (P00330) as an internal calibrant for absolute quantification.
